# Multi-omic insights into mitochondrial dysfunction and prostatic disease: evidence from transcriptomics, proteomics, and methylomics

**DOI:** 10.3389/fgene.2025.1609933

**Published:** 2025-08-22

**Authors:** Binbin Gong, Feixiang Yang, Ning Zhang, Zhengyang Wu, Tianrui Liu, Kun Wang, Xiangyu Zhang, Yangyang Zhang, Zhengyao Song, Chaozhao Liang

**Affiliations:** ^1^Department of Urology, The First Affiliated Hospital of Anhui Medical University, Hefei, China; ^2^Institute of Urology, Anhui Medical University, Hefei, China; ^3^Anhui Province Key Laboratory of Urological and Andrological Diseases Research and Medical Transformation, Anhui Medical University, Hefei, China; ^4^Department of Obstetrics and Gynecology, The First Affiliated Hospital of Anhui Medical University, Hefei, China; ^5^First School of Clinical Medicine, Anhui Medical University, Hefei, China; ^6^Second School of Clinical Medicine, Anhui Medical University, Hefei, China

**Keywords:** mitochondrial dysfunction, multi-omics, prostatitis, benign prostatic hyperplasia, prostate cancer

## Abstract

**Background:**

Prostatic diseases, consisting of prostatitis, benign prostatic hyperplasia (BPH), and prostate cancer (PCa), pose significant health challenges. While single-omics studies have provided valuable insights into the role of mitochondrial dysfunction in prostatic diseases, integrating multi-omics approaches is essential for uncovering disease mechanisms and identifying therapeutic targets.

**Methods:**

A genome-wide meta-analysis was conducted for prostatic diseases using the genome-wide association studies (GWAS) data from FinnGen and UK Biobank. Mitochondrial dysfunction-related genes were reviewed based on MitoCarta 3.0, with a library containing 1,244 mitochondrial genes. We integrated multi-omics through quantitative trait loci (QTL) across gene expression (eQTLs), protein abundance (pQTLs), and DNA methylation (mQTLs). We prioritized prostatic disease-related mitochondrial genes into three confidence tiers: Tier 1 (two eQTLs + pQTL + mQTL); Tier 2 (two eQTLs + pQTL/mQTL); and Tier 3 (eQTL + pQTL/mQTL). Further mediation analyses were performed to explore potential mediating pathways for the interaction between mitochondrial dysfunction and prostatic diseases, with 1,400 metabolomics and 731 immunomics.

**Results:**

We identified DCXR as the gene with Tier 1 evidence for BPH, validated by multi-omics integration through transcriptomic, proteomic, and methylomic signatures. We revealed two Tier 2 genes (NOA1 and ELAC2) and one Tier 3 gene (ACAT1) for BPH, two Tier 3 genes (TRMU and SFXN5) for prostatitis, and six Tier 3 genes (MRPL24, NDUFS6, PUS1, NBR1, GLOD4, and PCBD2) for PCa. We also explored the mediating pathways of mitochondrial genes (within the 3-tiers evidence) on prostatic diseases, and identified 8, 4, and 13 metabolites mediating the interaction between mitochondrial genes and BPH, prostatitis, and PCa, respectively, without the involvement of immune characters.

**Conclusion:**

These findings highlight the roles of mitochondrial dysfunction-related genes in prostatic diseases and identify key genes and pathways for potential therapeutic targets.

## 1 Introduction

Prostatic diseases, encompassing prostatitis, benign prostatic hyperplasia (BPH), and prostate cancer (PCa), bring challenges to global health. PCa is one of the major health burdens affecting middle-aged and elderly men, ranking as the third most common malignant tumor in males (Wu et al., 2024). BPH, characterized by non-cancerous enlargement of the prostate, affects nearly 50% of men aged over 50 years, with its prevalence rising with age (Chughtai et al., 2016). Prostatitis, including both chronic and acute forms, is associated with a marked decline in the quality of life, including daytime urinary frequency, urinary urgency, and even sexual dysfunction (Wagenlehner et al., 2013).

Although prostatic diseases are distinct clinical entities with different pathological features, they share overlapping risk factors and potentially interconnected pathogenic mechanisms, including chronic inflammation (Sfanos and De Marzo, 2012; Zlotta et al., 2014), hormonal imbalances (Tang et al., 2025), and cellular stress responses (Ouyang et al., 2005; Udensi and Tchounwou, 2016; Xu et al., 2025). Mitochondria are central to energy metabolism, providing ATP through oxidative phosphorylation (OXPHOS) and regulating metabolic processes like fatty acid oxidation and glucose metabolism (Horbinski and Chu, 2005). When dysfunctional, it leads to altered energy metabolism, increases reactive oxygen species (ROS) generation, and activation of oncogenic and inflammatory signaling pathways including PI3K/Akt and MAPK (Liu et al., 2018). Previous studies have suggested that mitochondrial-related pathways, such as altered OXPHOS and ROS production, play critical roles in the pathogenesis of prostatic diseases. For example, PCa cells exhibit a unique mitochondrial metabolic rewiring, shifting towards enhanced OXPHOS to support their rapid proliferation and aggressive behavior (Fontana et al., 2023). ROS-induced compensatory proliferation of prostate cells and associated inflammation are recognized as the pathogenic mechanisms underlying BPH and prostatitis (Xu et al., 2025; Minciullo et al., 2015).

Single-omic approaches, while valuable, often fail to provide a comprehensive understanding of complex diseases. Transcriptomics, proteomics, or methylomics alone cannot fully capture the multifaceted molecular alterations that drive disease progression. Multi-omics, integrating transcriptomics, proteomics, and methylomics provides a more holistic view of disease mechanisms. This complementary approach allows for better identification of disease biomarkers and therapeutic targets (Nativio et al., 2020). Despite advancements, key scientific questions remain unresolved, such as the exact interplay between mitochondrial dysfunction and the epigenetic alterations driving prostatic diseases. The boost of genome-wide association studies (GWAS) and quantitative trait loci (QTL) allows us to explore the molecular interaction through multi-omics. Here, we employed QTLs to assess relationships between mitochondrial dysfunction and prostatic diseases across transcriptomics, proteomics, and methylomics.

## 2 Methods

### 2.1 Data sources for prostatic diseases

The GWAS data for prostatic diseases were extracted from FinnGen study and UK Biobank (Table 1). Summary statistics of GWAS data for PCa were retrieved from the UK Biobank, which involved 183,888 participants, and from the FinnGen study, which included 146,465 individuals. For BPH, the data were obtained from UK Biobank (166,988 individuals) and FinnGen study (177,901 individuals). Prostatitis data were derived from the UK Biobank and FinnGen consortium, with sample sizes of 183,888 and 146,043 individuals, respectively. All participants were of European ancestry. A genome-wide meta-analysis was conducted on the independent GWASs of prostatic diseases using a fixed effects model implemented via METAL software (Willer et al., 2010).

**TABLE 1:** Detailed information on used studies.

Phenotype	Data source (consortium)	Sex	Sample size	Author; Year	PMID
Prostatic diseases					
Prostate cancer	Finngen	males	146,465	NA, 2022	NA
	UKBB	males	183,888	NA, 2018	NA
BPH	Finngen	males	177,901	NA, 2023	NA
	UKBB	males	166,988	NA, 2018	NA
prostatitis	Finngen	males	146,043	NA, 2023	NA
	UKBB	males	183,888	NA, 2018	NA
Multi-omics					
eqtl	eQTLGen	combined	31,684	NA, 2021	PMID: 34475573
	GTEx	combined	838	NA, 2020	PMID: 32913098
pQTL	deCODE	combined	35,559	Ferkingstad et al. (2021)	PMID:34857953
mQTL	LBC_BSGS_meta	combined	1,980	McRae et al. (2018); Wu et al. (2018)	PMID: 30514905; 29500431
Mediators					
Immune cells	Orrù et al. (2020)	combined	3,757	Orrù et al. (2020)	PMID:32929287
Plasma metabolites	Chen et al. (2023)	combined	7,659	Chen et al. (2023)	PMID: 36635386

### 2.2 Data sources of mitochondrial genes in multi-omics

Mitochondrial genes were obtained using MitoCarta3.0, with a library containing 1,244 mitochondrial genes (Rath et al., 2021). Quantitative trait loci (QTL) can uncover the relationships between single nucleotide polymorphisms (SNPs) and the levels of transcription, protein, and methylation. We extracted datasets of expression quantitative trait loci (eQTLs) from the eQTLGen (Võsa et al., 2021) and Genotype Tissue Expression (GTEx) (Consortium, 2020), protein quantitative trait loci (pQTLs) form the deCODE consortium (Ferkingstad et al., 2021), and methylation quantitative trait loci (mQTLs), from the LBC_BSGS_meta consortium (McRae et al., 2018) (Table 1). By screening mitochondrial genes, we identified 1,004 expressed genes from the eQTLGen consortium, 1,003 expressed genes from the GTEx consortium in prostate and whole blood tissues, 708 methylated genes with a total of 2,878 methylation sites from the LBC_BSGS_meta consortium, and 278 proteins from deCODE consortium with a threshold of P < 5 × 10^−5^.

### 2.3 Prioritizing prostatic disease-related mitochondrial genes

We estimate the correlation between mitochondrial genes and prostatic diseases at the gene expression, methylation, and protein abundance levels based on top-associated cis-QTLs using SMR analysis (Zhu et al., 2016; Liu et al., 2024). Windows centered on the respective genes (±1,000 kb) were considered, and SNPs were excluded at the threshold of P-value <5 × 10^−5^. To address genetic confounders, we incorporated the heterogeneity in dependent instruments (HEIDI) test to distinguish linkage disequilibrium from pleiotropy, where results exceeding the pleiotropy threshold (P-HEIDI <0.01) underwent rigorous quality control exclusion. Associations with the P-value <0.05 and P-HEIDI >0.01 were undertaken for further prioritization. Furthermore, we conducted colocalization analysis to detect shared causal variants between mitochondrial genes and prostatic diseases (Giambartolomei et al., 2014). This approach utilizes Bayesian statistical methods to estimate the probability of colocalization, thereby assessing the likelihood that the same genetic variants influence both phenotypes (Yoshiji et al., 2023; Battle et al., 2017). A posterior probability of H4 (PPH4) > 0.70 or H3 (PPH3) + H4 (PPH4) > 0.70 was regarded as evidence of colocalization (Chen et al., 2024).

### 2.4 Integration of evidence from multi-omics level

**FIGURE 1:**
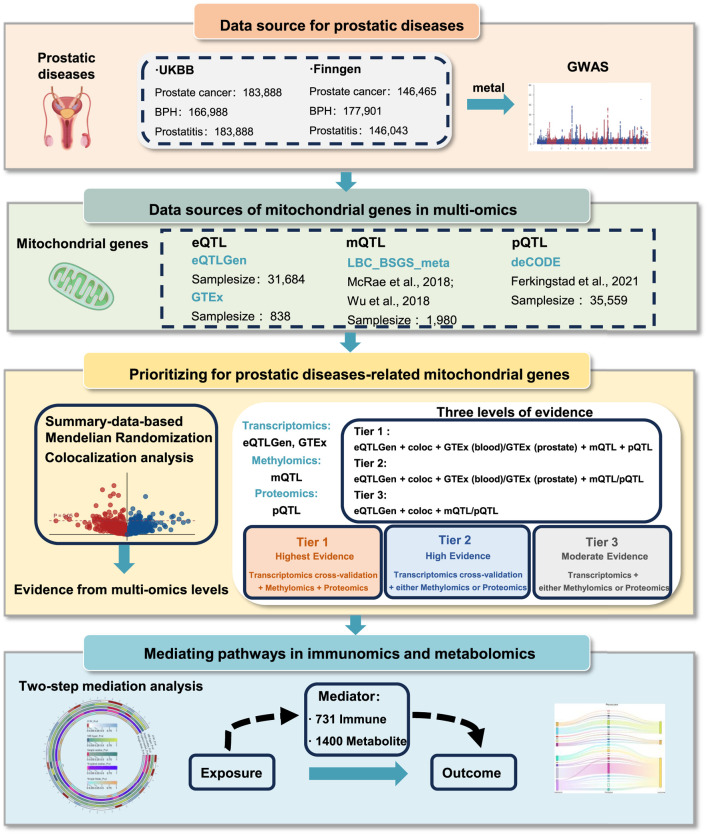
Overall study design.

We integrated these results from distinct molecular levels. To address the interpretational constraints of single-omic approaches, cross-validation through at least two omics layers was mandatory for each candidate gene in our tri-layered classification. We classified the candidate genes into three tiers (Figure 1): Tier 1) Tier 1 genes are defined as those associated with prostatic diseases at the cross-validation of transcriptomics both in eQTLgen consortium and GTEx consortium (P-value <0.05), with positive posterior probability in colocalization analysis, and showing associations with prostatic diseases at both methylation and protein levels (P-value <0.05). Tier 2) Tier 2 genes are regarded as those associated with prostatic diseases at the level of gene expression both in eQTLgen consortium and GTEx tissues (P-value <0.05), with evidence of colocalization, and presenting positive evidence either the protein or methylation level (P-value <0.05). Tier 3) Tier 3 genes are defined as those associated with prostatic diseases at the gene expression level in eQTLgen (P-value <0.05), with co-localized evidence, and positive evidence at either the protein or methylation levels (P-value <0.05).

### 2.5 Validation of prioritized genes through RNA-sequencing

To further validate the prioritized genes, we explored the expression patterns between normal and tumor, hyperplasia, and inflammation samples of prostate through RNA-sequencing data. We extracted two independent datasets for BPH (GSE7307 and GSE132714) and PCa (GSE46602 and GSE70768) from Gene Expression Omnibus (GEO) database. Due to the unavailability of human prostate tissue sequencing data from prostatitis patients, we utilized the murine prostate tissue RNA-sequencing data from established prostatitis mouse models at Anhui Medical University (Zhang et al., 2022).

### 2.6 Mediating pathways in immunomics and metabolomics

To explore the biological mechanisms underlying these genetic associations, we conducted a mediation analysis with 1,400 metabolomics, and 731 immunomics. A two-step mediation analysis was conducted to evaluate the potential mediating effects of prioritized mitochondrial gene-related protein, metabolite, and immune characteristics on the causal associations between mitochondrial genes and prostatic diseases, with the basis of TwoSampleMR R package. Specifically, we initially estimated the causal effects of mitochondrial genes on potential mediators (metabolomics and immunomics), and we subsequently measured the effects of mediators on prostatic diseases. The mediation effects were calculated through the “product of coefficients” method, namely, *mediation effect* = *mediation effect A* * *mediation effect B*, the *mediation effect A* refers to the estimates in the first step, and the *mediation effect B* refers to the estimates in the second step. Total effect meant the effect of mitochondrial genes on prostatic diseases. Only mediators whose effect directions of total effect and mediation effect were consistent were included in the mediation analysis.

## 3 Result

### 3.1 Identifying BPH-related mitochondrial genes in multi-omics

**FIGURE 2:**
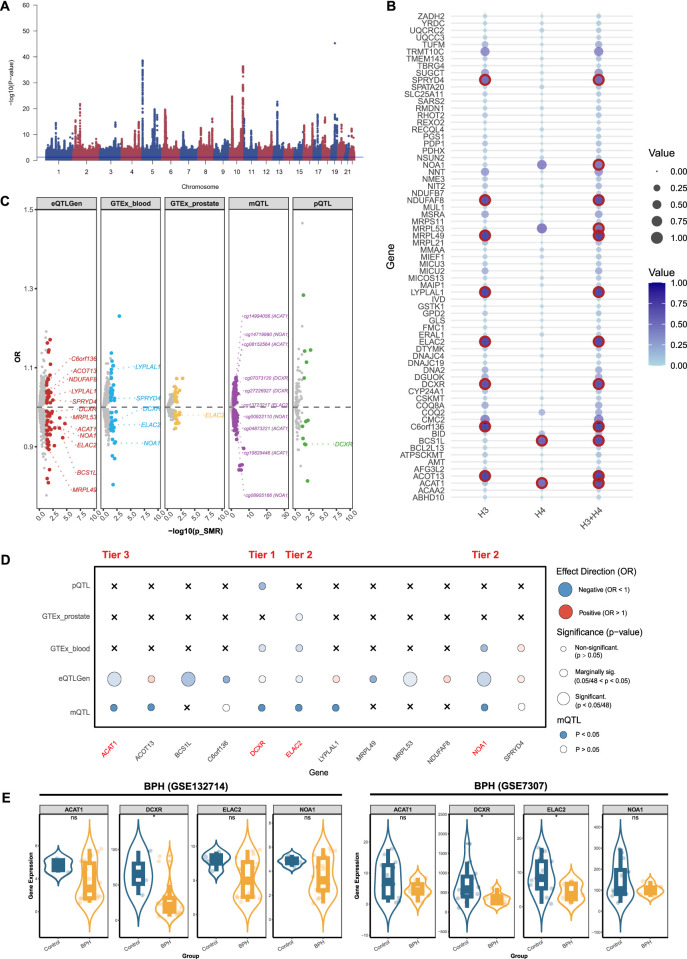
Prioritizing for benign prostatic hyperplasia (BPH)-related mitochondrial genes through evidence from transcriptomics, methylomics, and proteomics. **(A)** Manhattan plot for the genome-wide meta-analysis of BPH. **(B)** Bubble plots for the results from colocalization analysis. Genes highlighted in red indicate significant results. **(C)** BPH-related mitochondrial genes in the transcriptome (eQTLGen and GTEx), methylome (LBC_BSGS), and proteome (deCODE) based on Summary data-based Mendelian randomization (SMR). Genes with evidence of colocalization are prominently marked in the figure. **(D)** Prioritizing for BPH-related mitochondrial genes through evidence from transcriptomics, proteomics, and methylomics. **(E)** Validation of prioritized genes through RNA-sequencing. *: P-value <0.05; **: P-value <0.01; ***: P-value <0.001.

We conducted a genome-wide meta-analysis to aggregate the GWAS data of BPH from UK Biobank and FinnGen. Multivariate GWAS identified 61 loci and 7,756 SNPs (P-value <5 × 10^−8^) associated with the genic architecture of BPH, and the Manhattan plot of the GWAS was shown in Figure 2A. We conducted multi-omics analysis using the eQTLs, mQTLs, and pQTLs databases. Initially, based on the eQTLGen database, we identified 69 significant genes (Supplementary Figure S1A; Supplementary Table S1), 12 of which were validated by colocalization (Figures 2B,C). Furthermore, through the GTEx database, we identified 40 positive genes in blood tissue and 20 positive genes in prostate tissue ((Supplementary Figure S1B,C; Supplementary Table S2,3). Among the 12 genes with colocalization evidence, only LYPLAL1, SPRYD4, DCXR, ELAC2, and NOA1 in blood tissue, and ELAC2 in prostate tissue showed significant evidence (Figure 2C). In addition, we identified 12 proteins related to BPH via the deCODE database ((Supplementary Figure S1D; Supplementary Table S4), of which only the DCXR presented the colocalization evidence (Figure 2C). Using the mQTL database, we identified 216 methylation sites ((Supplementary Figure S1E; Supplementary Table S5), 10 of them showed the evidence of colocalization (ACAT1: cg14994056, cg08152564, cg04873221, cg19829446; NOA1: cg14719990, cg00922110, cg08905166; DCXR: cg07073120, cg27226927; ELAC2: cg13723217) (Figure 2C). After consolidating the multi-omics analysis results, we identified one tier 1 gene (DCXR), two tier 2 genes (NOA1 and ELAC2), and one tier 3 gene (ACAT1) (Figure 2D).

### 3.2 Identifying prostatitis-related mitochondrial genes in multi-omics

**FIGURE 3:**
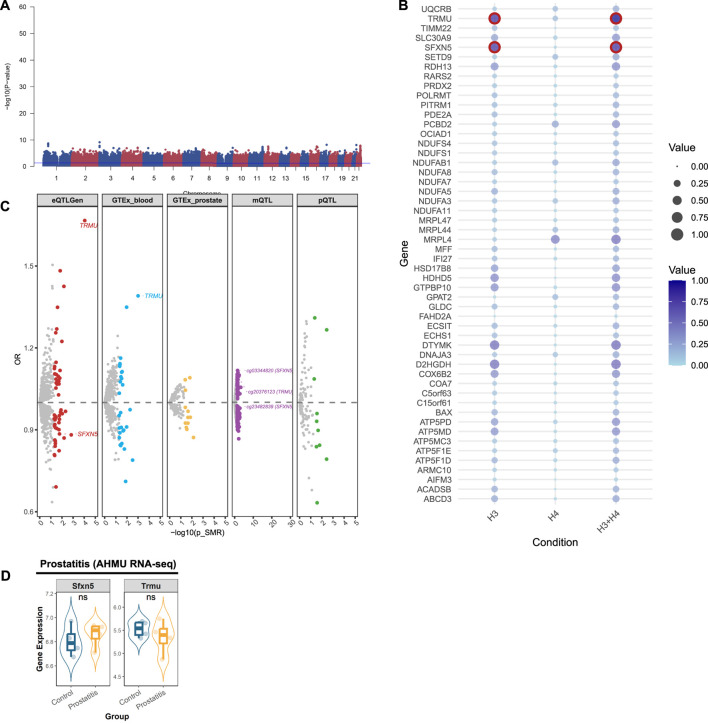
Prioritizing for prostatitis-related mitochondrial genes through evidence from transcriptomics, methylomics, and proteomics. **(A)** Manhattan plot for the genome-wide meta-analysis of prostatitis. **(B)** Bubble plots for the results from colocalization analysis. Genes highlighted in red indicate significant results. **(C)** Prostatitis-related mitochondrial genes in the transcriptome (eQTLGen and GTEx), methylome (LBC_BSGS), and proteome (deCODE) based on Summary data-based Mendelian randomization (SMR). Genes with evidence of colocalization are prominently marked in the figure. **(D)** Validation of prioritized genes through RNA-sequencing. *: P-value <0.05; **: P-value <0.01; ***: P-value <0.001.

Multivariate GWAS identified 6 loci and 44 SNPs (P-value <5 × 10^−8^) associated with the genetic inheritance of prostatitis (Figure 3A). We further analyzed mitochondrial genes related to prostatitis. Initially, using the eQTLGen database, we identified 52 significant genes (Supplementary Figure S2A; Supplementary Table S6), 2 of which were validated by colocalization (Figure 3B). Furthermore, through the GTEX database, we identified 26 positive genes in blood tissue and 6 positive genes in prostate tissue (Supplementary Figure S2B,C; Supplementary Table S7,8). Among them, only TRMU in blood tissue presented evidence of colocalization (Figure 3C). In addition, we identified 10 proteins related to prostatitis via the deCODE database (Supplementary Figure S2D; Supplementary Table S9), although none showed colocalized evidence (Figure 3C). Using the mQTL database, we identified 118 methylation sites (Supplementary Figure S2E; Supplementary Table S10), of which only 3 sites presented colocalization evidence (SFXN5: cg03344820, cg23482839; TRMU: cg20376123). After consolidating the multi-omics analysis results, we identified two tier 3 genes (TRMU and SFXN5) (Figure 3C).

### 3.3 Identifying PCa-related mitochondrial genes in multi-omics

**FIGURE 4:**
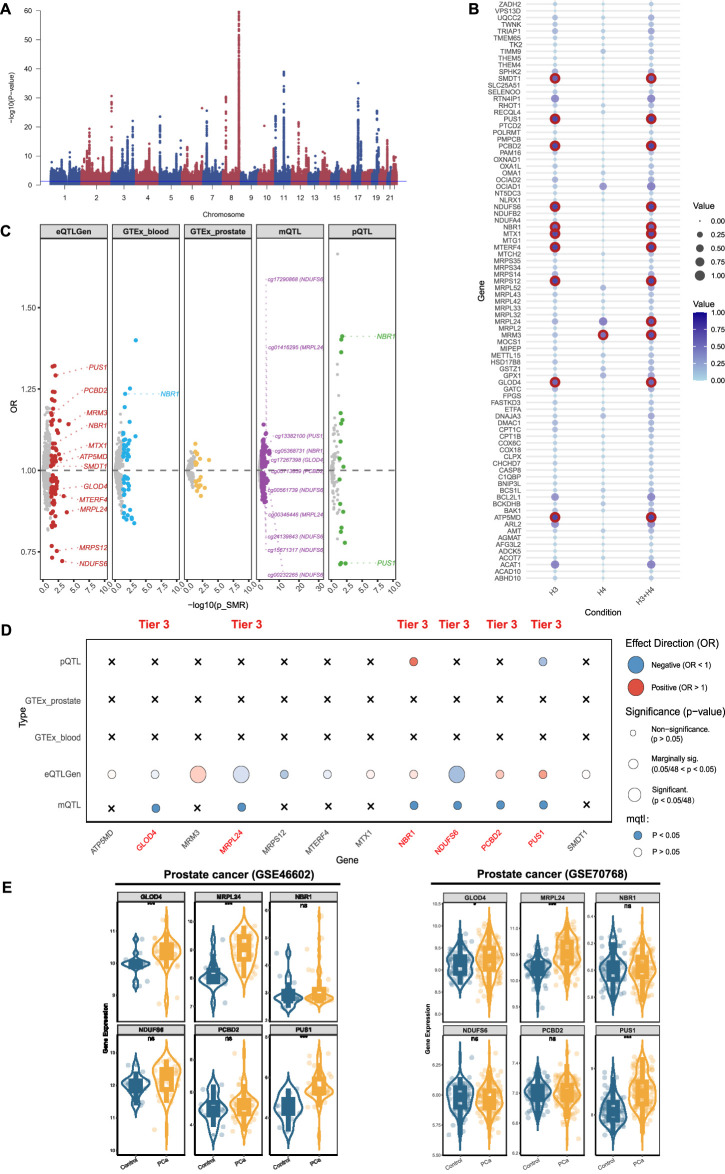
Prioritizing for prostate cancer (PCa)-related mitochondrial genes through evidence from transcriptomics, methylomics, and proteomics. **(A)** Manhattan plot for the genome-wide meta-analysis of PCa. **(B)** Bubble plots for the results from colocalization analysis. Genes highlighted in red indicate significant results. **(C)** PCa-related mitochondrial genes in the transcriptome (eQTLGen and GTEx), methylome (LBC_BSGS), and proteome (deCODE) based on Summary data-based Mendelian randomization (SMR). Genes with evidence of colocalization are prominently marked in the figure. **(D)** Prioritizing for PCa-related mitochondrial genes through evidence from transcriptomics, proteomics, and methylomics. **(E)** Validation of prioritized genes through RNA-sequencing. *: P-value <0.05; **: P-value <0.01; ***: P-value <0.001.

For PCa, genome-wide meta-analysis identified 64 loci and 5,011 SNPs (P-value <5 × 10^−8^) (Figure 4A). For mitochondrial genes associated with PCa, we identified 86 significant genes using the eQTLGen database (Supplementary Figure S3A; Supplementary Table S11), 12 of which passed colocalization validation (Figure 4B). Furthermore, through the GTEX database, we identified 41 positive genes in blood and 17 positive genes in prostate tissue (Supplementary Figure S3B,C; Supplementary Tables S12, 13). Among them, only NBR1 in blood tissue presented colocalized evidence (Figure 4C). Using the deCODE database, we identified 16 proteins related to PCa (Supplementary Figure S2D; Supplementary Table S14), of which NBR1 and PUS1 showed the evidence of colocalization (Figure 4C). Through the mQTL database, we identified 270 methylation sites, (Supplementary Figure S2E; Supplementary Table S15). Among them, 11 sites were selected (NDUFS6: cg17290868, cg00561739, cg24139843, cg15671317, cg00232265; PUS1: cg13382100; NBR1: cg05368731; GLOD4: cg17267398; PCBD2: cg05713859) (Figure 4C). After summarizing the multi-omics analysis results, we identified 6 tier 3 genes: MRPL24, NDUFS6, PUS1, NBR1, GLOD4, and PCBD2 (Figure 4D).

### 3.4 Prioritizing prostatic disease-related mitochondrial genes

After integrating the multi-omics evidence, we prioritized one gene with tier 1 evidence, two genes with tier 2 evidence, and nine genes with tier 3 evidence associated with prostatic diseases (Table 2). Notably, all significant mitochondrial signatures presented a consistent effect direction both in gene expression (eQTLGen and GTEx datasets) and protein levels, while some methylation sites showed different directions. Among them, DCXR was identified as a tier 1 gene related to BPH, which was validated in transcriptomics, proteomics, and methylomics across three dimensions. In the results from eQTLGen, it was identified as a protective gene [OR (95% CI) = 0.99 (0.98, 1.00), P-value = 0.03] and was validated in the colocalization analysis (PP.H3 + PP.H4 = 0.951). It was also found to be a protective effect in both GTEx [OR (95% CI) = 0.96 (0.93, 1.00), P-value = 0.03] and pQTL [OR (95% CI) = 0.907 (0.835, 0.984), P-value = 0.02]. Moreover, we identified two key methylation sites for DXCR, cg07073120 [OR (95% CI) = 1.04 (1.01, 1.08), P-value = 0.03] and cg27226927 [OR (95% CI) = 1.01 (1.00, 1.02), P-value = 0.03], despite the inconsistency in their effect directions compared to the previous analysis. NOA1 and ELAC2 were identified as a tier 2 genes related to BPH, exhibiting protective effects both in eQTL and pQTL levels. For prostatitis, TRMU and SFXN5 were identified as tier 3 genes, with TRMU emerging as a risk gene [eQTLGen: OR (95% CI) = 1.67 (1.29, 2.15), P-value = 8.96 × 10^−5^], and SFXN5 as a protective factor [eQTLGen: OR (95% CI) = 0.89 (0.80, 0.98), P-value = 0.02]. We further prioritized six tier 3 genes associated with PCa, of which MRPL24, NDUFS6, and GLOD4 were related to decreased risk of PCa, and PUS1, NBR1, and PCBD2 were related to increased risk.

**TABLE 2:** Tier of candidate genes for prostatic diseases with multi-omics analyses.

Outcome gene tier			eQTLGen					GTEx (whole blood)		GTEx (prostate)		pQTL		mQTL		
			Or (95% CI)	P-value	PP.H3	PP.H4	PP.H3+PP.H4	Or (95% CI)	P-value	Or (95% CI)	P-value	Or (95% CI)	P-value	Probe	Or (95% CI)	P-value
BPH	DCXR	Tier 1	0.99 (0.98, 1.00)	0.03	0.950	1.44E-03	0.951	0.96 (0.93, 1.00)	0.03			0.91 (0.84, 0.98)	0.02	cg07073120	1.04 (1.01, 1.08)	0.03
														cg27226927	1.01 (1.00, 1.02)	0.03
	NOA1	Tier 2	0.95 (0.92, 0.98)	2.19E-04	0.102	0.605	0.707	0.91 (0.85, 0.98)	9.11E-03					cg00922110	0.98 (0.97, 0.99)	3.63E-04
														cg08905166	0.94 (0.91, 0.98)	8.57E-04
														cg14719990	1.07 (1.03, 1.11)	1.08E-03
	ELAC2		0.98 (0.96, 1.00)	0.03	0.990	1.79E-04	0.990	0.96 (0.92, 1.00)	0.04	0.98 (0.97, 1.00)	0.01			cg13723217	1.01 (1.00, 1.01)	0.04
	ACAT1	Tier 3	0.96 (0.94, 0.98)	2.21E-05	0.178	0.778	0.956							cg04873221	0.98 (0.97, 0.99)	4.16E-04
														cg19829446	0.97 (0.96, 0.99)	4.18E-04
														cg08152564	1.07 (1.03, 1.11)	1.72E-03
														cg14994056	1.08 (1.03, 1.13)	2.19E-03
Prostatitis	TRMU	Tier 3	1.67 (1.29, 2.15)	8.96E-05	0.861	0.138	0.999							cg20376123	1.10 (1.01, 1.19)	0.02
	SFXN5		0.89 (0.80, 0.98)	0.02	0.826	0.022	0.848							cg23482839	0.98 (0.97, 1.00)	0.01
														cg03344820	1.12 (1.01, 1.23)	0.03
Prostate cancer	MRPL24	Tier 3	0.87 (0.81, 0.94)	1.36E-04	0.299	0.629	0.928							cg01416295	1.07 (1.03, 1.11)	3.54E-04
														cg00346446	0.90 (0.86, 0.96)	4.61E-04
	NDUFS6		0.72 (0.60, 0.87)	7.56E-04	0.999	5.97E-06	0.999							cg00232265	0.97 (0.95, 0.98)	5.20E-05
														cg17290868	1.11 (1.05, 1.18)	3.65E-04
														cg15671317	0.92 (0.87, 0.97)	9.34E-04
														cg00561739	0.93 (0.89, 0.97)	1.33E-03
														cg24139843	0.90 (0.85, 0.96)	2.07E-03
	GLOD4		0.94 (0.89, 1.00)	0.03	0.835	5.95E-03	0.841							cg17267398	0.98 (0.97, 1.00)	0.02
	PUS1		1.29 (1.07, 1.57)	8.94E-03	0.999	3.09E-05	0.999					0.72 (0.53, 0.96)	0.03	cg13382100	1.04 (1.01, 1.07)	0.01
	NBR1		1.08 (1.02, 1.15)	0.01	0.880	7.56E-03	0.888					1.41 (1.06, 1.88)	0.02	cg05368731	0.99 (0.98, 1.00)	7.83E-03
	PCBD2		1.17 (1.01, 1.36)	0.04	1.000	3.77E-06	1.000							cg05713859	0.97 (0.95, 1.00)	0.04

We validated theses prioritized genes through RNA-sequencing data. For those 4 genes in BPH, only DCXR was cross-validated in two datasets (GSE132714: P-value <0.05; GSE7307: P-value <0.05), while ELAC2 was validated in GSE7307 (P-value <0.05) (Figure 2E). Neither of the two candidate genes in prostatitis presented supporting evidence from RNA-sequencing (Figure 3D). For 6 prioritized genes in PCa, GLOD4 (GSE46602: P-value <0.001; GSE70768: P-value <0.05), MRPL24 (GSE46602: P-value <0.001; GSE70768: P-value <0.001), and PUS1 (GSE46602: P-value <0.001; GSE70768: P-value <0.001) were cross-validated in two RNA-sequencing datasets (Figure 4E).

### 3.5 Mediating pathways in immunomics and metabolomics

**FIGURE 5:**
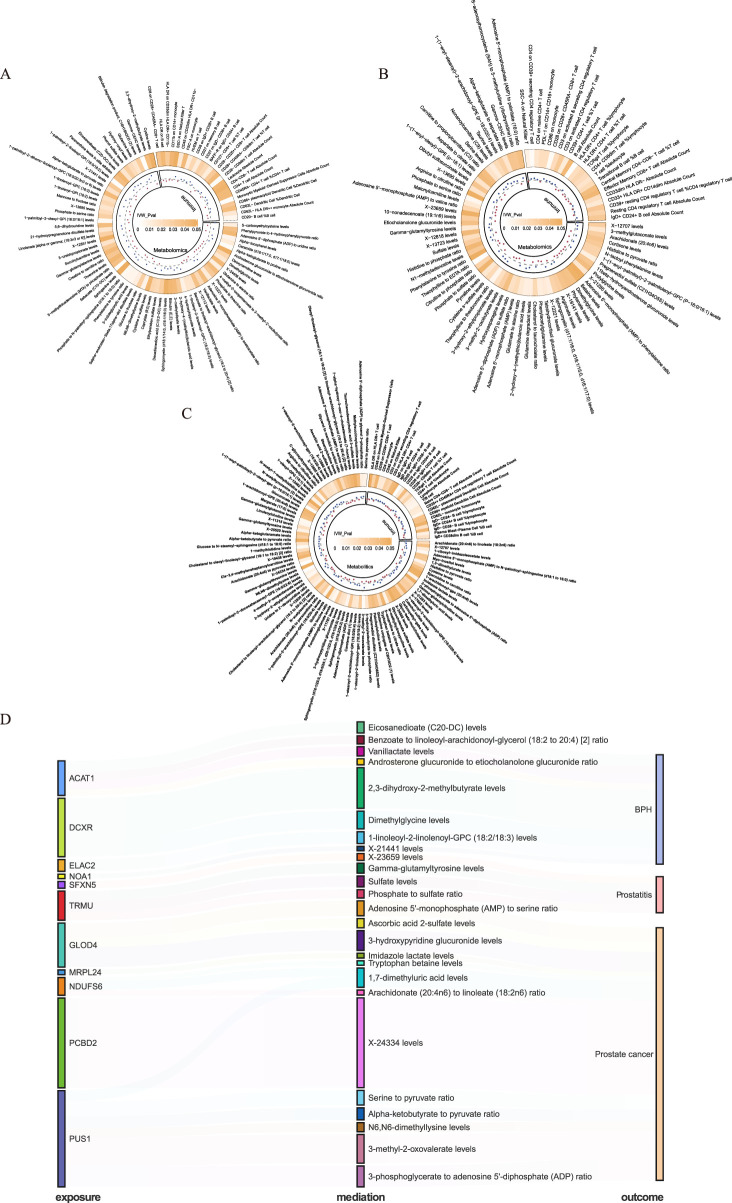
Mediating pathways between mitochondrial genes and prostatic diseases in immunomics and metabolomics. **(A)** Benign prostatic hyperplasia (BPH)-related immune and metabolic signatures. **(B)** Prostatitis-related immune and metabolic signatures. **(C)** Prostate cancer (PCa)-related immune and metabolic signatures. **(D)** Mediating pathways between mitochondrial genes and prostatic diseases in metabolomics.

We performed mediation analyses to identify metabolomic, and immunologic mediators linking mitochondrial dysfunction to prostatic diseases. Screening 731 immune traits, and 1,400 circulating metabolites, we identified 25 immune features and 66 metabolites associated with BPH (Figure 5A; Supplementary Table S16), and, 23 immune features, and 57 metabolites linked to prostatitis (Figure 5B; Supplementary Table S17). Besides, we identified 23 immune features and 77 metabolites associated with PCa (Figure 5C; Supplementary Table S18). We also explored the causal effects of mitochondrial genes (within the 3-tiers evidence) on significant immune and metabolic features (Supplementary Table S19–21), and conducted a two-step analysis to estimate the mediation effects (Supplementary Table S22–24). We found 8, 4, and 13 metabolites mediating the interaction between mitochondrial genes and BPH, prostatitis, and PCa, respectively, without the involvement of immune characters (Figure 5D). Among them, 2,3-dihydroxy-2-methyl-butyrate levels exhibited a significant mediation effect linking the DCXR gene and BPH with a mediation proportion of 22.0%, and the level of 3-methyl-2-oxovalerate linking PUS1 and PCa with mediation proportion of 15.5%, sulfate levels mediated association between TRMU and prostatitis with 5.9% (Supplementary Table S22–24).

## 4 Discussion

In the current study, we identified prostatic disease-related mitochondrial signatures through evidence from transcriptomics, proteomics, and methylomics. Notably, DCXR emerged as the gene with Tier 1 evidence for BPH. Our analysis revealed two Tier 2 genes (NOA1 and ELAC2) for BPH, two Tier 3 genes (TRMU and SFXN5) for prostatitis, and six Tier 3 genes (MRPL24, NDUFS6, PUS1, NBR1, GLOD4, and PCBD2) for PCa. Through metabolic mediation analysis, we uncovered specific metabolic pathways through which these genes might influence disease development. BPH-associated genes showed strong connections with dimethylglycine and eicosanedioate metabolism, while prostatitis-related genes were linked to sulfate and phosphate pathways. PCa-associated genes demonstrated diverse metabolic mediations, including amino acid and lipid metabolism. These findings provide novel insights into the distinct molecular mechanisms underlying these prostatic diseases.

The multi-omics integration revealed critical genes with disease-specific mechanistic implications. DCXR, identified as a tier-1 gene in BPH. DCXR has been reported to promote the proliferation and cell cycle progression of breast cancer by promoting glycolysis activity (Cho-Vega et al., 2007). While DCXR is known to be upregulated in prostate cancer and expressed at low levels in normal prostate epithelial cells (Cho-Vega et al., 2007), its role in BPH has not been extensively explored. DCXR, dicarbonyl/L-xylulose reductase, encodes an enzyme involved in ketone body metabolism and the detoxification of reactive dicarbonyl compounds, which are known to cause cellular damage through protein glycation and oxidative stress (Lee et al., 2013). It may reduce prostate cell damage and compensatory hyperplasia by decreasing oxidative stress (Udensi and Tchounwou, 2016). The DCXR presented the protective factor in chronic kidney diseases for its function of dicarbonyls clearance (Perco et al., 2019). An in-depth study of the functional mechanism of DCXR in BPH may help to elucidate the molecular basis of the disease and advance the development of precision therapeutic strategies. SFXN5, a mitochondrial protein predicted to enable citrate transmembrane transporter activity (Lockhart et al., 2002), was identified as a prostatitis-related gene. It was found that the elevated expression in neuroendocrinal prostate cancers, with low expression in normal prostate tissues (Huang et al., 2025). Functionally, the deficiency of SFXN5 impairs neutrophil recruitment and spreading, highlighting its role in inflammation and immune response (Zhang et al., 2023). This mitochondrial-immune axis could be pivotal in driving or sustaining the inflammatory processes observed in prostatitis. Understanding how SFXN5 modulates immune cell behavior and mitochondrial metabolism may therefore provide novel insights into prostatitis pathogenesis and uncover potential therapeutic targets aimed at mitigating inflammation. For the PCa-related genes, mitochondrial ribosomal protein L24 (MRPL24) and NADH: ubiquinone oxidoreductase subunit S6 (NDUFS6) converge on mitochondrial energy regulation. MRPL24, crucial for mitochondrial translation, could influence cellular metabolism and energy production, potentially affecting tumor growth and survival (Di Nottia et al., 2020). NDUFS6, part of mitochondrial respiratory chain complex I, impacts mitochondrial function and may modulate immune surveillance and tumor growth when targeted (Liang et al., 2025).

Notably, the results of DCXR from transcriptomics and methylomics were diverse in the effect direction. The higher expression of DCXR was associated with decreased risk of BPH, while hypermethylation of cg07073120 was related to a lower risk. DNA methylation patterns vary across different genomic contexts. For instance, promoter methylation generally represses gene expression, while methylation within exonic regions may influence alternative splicing or transcript stability (Jones, 2012). Moreover, the regulatory effects of DNA methylation are often tissue- and cell type-specific (Jones, 2012). Based on the UCSC Genome Browser (https://genome.ucsc.edu/), cg07073120 (chr17:79994884) was located in the exonic regions of DCXR (ch17:79993734-79995573), which may inhibit its expression.

Through mediation analysis, we identified potential metabolic mediators that delineate branched-chain amino acid (BCAA) metabolism (2,3-dihydroxy-2-methyl-butyrate and 3-methyl-2-oxovalerate levels) and sulfur metabolic response (sulfate levels) as potential pathways linking exposures to prostatic diseases. Specifically, 2,3-dihydroxy-2-methyl-butyrate levels mediated the association between the DCXR gene and BPH, while 3-methyl-2-oxovalerate levels mediated the effect of PUS1 on PCa. 2,3-dihydroxy-2-methylbutyrate, identified as an intermediate of valine catabolism (Biliotti et al., 2021), jointly participates in BCAA metabolism with the classic BCAA metabolite 3-methyl-2-oxovalerate (Fulcher et al., 2022). Inhibited branched-chain amino acid catabolism, such as valine, has been reported to be associated with impaired cellular mitochondrial respiration and reduced prostate cell proliferation (Bidgood et al., 2024). Furthermore, sulfate levels mediated the association between TRMU and prostatitis. Sulfate-related products participate in the biological processes of prostatic diseases. For instance, dehydroepiandrosterone sulfate (DHEA-S), a hormone precursor, is linked to the risk of chronic prostatitis (Vakina et al., 2003), and enhanced dehydroepiandrosterone (DHEA) metabolism sustains the progression of castration-resistant PCa (Chang et al., 2011). These metabolic pathways may represent important links between mitochondrial dysfunction and prostatic diseases.

In this study, we combined evidence from multi-omic levels to strengthen the relationships between mitochondrial genes and prostate diseases. There are also some limitations in our study. Given the limited number of mitochondria-associated proteins in the deCODE consortium, some prostatic disease-related mitochondrial proteins may be ignored. In addition, associations of gene expression and protein levels were consistent with disease risk regulation, whereas some methylation effects exhibited site-specific heterogeneity, indicating the complexity of epigenetic regulation. Another limitation of this study is that the posterior probability of colocalization warrants careful interpretation, in cases where PPH3 is small, a low PP4 may not imply the absence of colocalization. Notably, due to the unavailability of human RNA-sequencing data for prostatitis, we used results from mice, where interspecies differences may introduce discrepancies in gene expression.

In conclusion, our comprehensive multi-omics analysis has identified key mitochondrial genes associated with prostatic diseases. We prioritized genes with multi-omics levels of evidence, highlighting DCXR as the tier 1 gene for BPH. Additionally, our mediation analysis revealed significant immune and metabolite features linked to these conditions, suggesting potential pathways through which mitochondrial dysfunction influences disease risk. These findings emphasize the importance of multi-omics integration in unraveling disease mechanisms and identifying therapeutic targets. Providing a foundation for further investigation into the molecular mechanisms underlying prostatic diseases and may inform the development of novel therapeutic strategies.

## Data Availability

The original contributions presented in the study are included in the article/Supplementary Material, further inquiries can be directed to the corresponding author.
