# Supplementary material for: Multi-omic insights into mitochondrial dysfunction and prostatic disease: evidence from transcriptomics, proteomics, and methylomics
Source: Front Genet. 2025 Aug 22;16:1609933. doi: 10.3389/fgene.2025.1609933 (PMC12411192; doi:10.3389/fgene.2025.1609933)
Supplement: Supplementary file 1 [file Table2.docx]

**Table1.** Detailed information on used studies

| **Phenotype** | **Data source (consortium)** | **Sex** | **Sample size** | **Author; Year** | **PMID** |
| --- | --- | --- | --- | --- | --- |
| Prostatic diseases |  |  |  |  |  |
| Prostate cancer | Finngen | males | 146,465 | NA, 2022 | NA |
|  | UKBB | males | 183,888 | NA, 2018 | NA |
| BPH | Finngen | males | 177,901 | NA, 2023 | NA |
|  | UKBB | males | 166,988 | NA, 2018 | NA |
| prostatitis | Finngen | males | 146,043 | NA, 2023 | NA |
|  | UKBB | males | 183,888 | NA, 2018 | NA |
| Multi-omics |  |  |  |  |  |
| eqtl | eQTLGen | combined | 31,684 | NA, 2021 | PMID: 34475573 |
|  | GTEx | combined | 838 | NA, 2020 | PMID: 32913098 |
| pQTL | deCODE | combined | 35,559 | Ferkingstad et al., 2021 | PMID: 34857953 |
| mQTL | LBC_BSGS_meta | combined | 1,980 | McRae et al., 2018; Wu et al., 2018 | PMID: 30514905; 29500431 |
| Mediators |  |  |  |  |  |
| Immune cells | Orrù et al.; 2020 | combined | 3,757 | Orrù et al.; 2020 | PMID: 32929287 |
| Plasma metabolites | Chen et al.; 2023 | combined | 7,659 | Chen et al.; 2023 | PMID: 36635386 |
