# Supplementary material for: Multi-omic insights into mitochondrial dysfunction and prostatic disease: evidence from transcriptomics, proteomics, and methylomics
Source: Front Genet. 2025 Aug 22;16:1609933. doi: 10.3389/fgene.2025.1609933 (PMC12411192; doi:10.3389/fgene.2025.1609933)
Supplement: Supplementary file 2 [file Table3.docx]

**Table2.** Tier of candidate genes for prostatic diseases with multi-omics analyses

| Outcome  Gene  Tier | | | eQTLGen | | | | | GTEx (whole blood) | | GTEx (prostate) | | pQTL | | mQTL | | |
| --- | --- | --- | --- | --- | --- | --- | --- | --- | --- | --- | --- | --- | --- | --- | --- | --- |
|  |  |  | OR (95% CI) | P-value | PP.H3 | PP.H4 | PP.H3+PP.H4 | OR (95% CI) | P-value | OR (95% CI) | P-value | OR (95% CI) | P-value | Probe | OR (95% CI) | P-value |
| BPH | DCXR | Tier 1 | 0.99 (0.98, 1.00) | 0.03 | 0.950 | 1.44E-03 | 0.951 | 0.96 (0.93, 1.00) | 0.03 |  |  | 0.91 (0.84, 0.98) | 0.02 | cg07073120 | 1.04 (1.01, 1.08) | 0.03 |
|  |  |  |  |  |  |  |  |  |  |  |  |  |  | cg27226927 | 1.01 (1.00, 1.02) | 0.03 |
|  | NOA1 | Tier 2 | 0.95 (0.92, 0.98) | 2.19E-04 | 0.102 | 0.605 | 0.707 | 0.91 (0.85, 0.98) | 9.11E-03 |  |  |  |  | cg00922110 | 0.98 (0.97, 0.99) | 3.63E-04 |
|  |  |  |  |  |  |  |  |  |  |  |  |  |  | cg08905166 | 0.94 (0.91, 0.98) | 8.57E-04 |
|  |  |  |  |  |  |  |  |  |  |  |  |  |  | cg14719990 | 1.07 (1.03, 1.11) | 1.08E-03 |
|  | ELAC2 |  | 0.98 (0.96, 1.00) | 0.03 | 0.990 | 1.79E-04 | 0.990 | 0.96 (0.92, 1.00) | 0.04 | 0.98 (0.97, 1.00) | 0.01 |  |  | cg13723217 | 1.01 (1.00, 1.01) | 0.04 |
|  | ACAT1 | Tier 3 | 0.96 (0.94, 0.98) | 2.21E-05 | 0.178 | 0.778 | 0.956 |  |  |  |  |  |  | cg04873221 | 0.98 (0.97, 0.99) | 4.16E-04 |
|  |  |  |  |  |  |  |  |  |  |  |  |  |  | cg19829446 | 0.97 (0.96, 0.99) | 4.18E-04 |
|  |  |  |  |  |  |  |  |  |  |  |  |  |  | cg08152564 | 1.07 (1.03, 1.11) | 1.72E-03 |
|  |  |  |  |  |  |  |  |  |  |  |  |  |  | cg14994056 | 1.08 (1.03, 1.13) | 2.19E-03 |
| Prostatitis | TRMU | Tier 3 | 1.67 (1.29, 2.15) | 8.96E-05 | 0.861 | 0.138 | 0.999 |  |  |  |  |  |  | cg20376123 | 1.10 (1.01, 1.19) | 0.02 |
|  | SFXN5 |  | 0.89 (0.80, 0.98) | 0.02 | 0.826 | 0.022 | 0.848 |  |  |  |  |  |  | cg23482839 | 0.98 (0.97, 1.00) | 0.01 |
|  |  |  |  |  |  |  |  |  |  |  |  |  |  | cg03344820 | 1.12 (1.01, 1.23) | 0.03 |
| Prostate cancer | MRPL24 | Tier 3 | 0.87 (0.81, 0.94) | 1.36E-04 | 0.299 | 0.629 | 0.928 |  |  |  |  |  |  | cg01416295 | 1.07 (1.03, 1.11) | 3.54E-04 |
|  |  |  |  |  |  |  |  |  |  |  |  |  |  | cg00346446 | 0.90 (0.86, 0.96) | 4.61E-04 |
|  | NDUFS6 |  | 0.72 (0.60, 0.87) | 7.56E-04 | 0.999 | 5.97E-06 | 0.999 |  |  |  |  |  |  | cg00232265 | 0.97 (0.95, 0.98) | 5.20E-05 |
|  |  |  |  |  |  |  |  |  |  |  |  |  |  | cg17290868 | 1.11 (1.05, 1.18) | 3.65E-04 |
|  |  |  |  |  |  |  |  |  |  |  |  |  |  | cg15671317 | 0.92 (0.87, 0.97) | 9.34E-04 |
|  |  |  |  |  |  |  |  |  |  |  |  |  |  | cg00561739 | 0.93 (0.89, 0.97) | 1.33E-03 |
|  |  |  |  |  |  |  |  |  |  |  |  |  |  | cg24139843 | 0.90 (0.85, 0.96) | 2.07E-03 |
|  | GLOD4 |  | 0.94 (0.89, 1.00) | 0.03 | 0.835 | 5.95E-03 | 0.841 |  |  |  |  |  |  | cg17267398 | 0.98 (0.97, 1.00) | 0.02 |
|  | PUS1 |  | 1.29 (1.07, 1.57) | 8.94E-03 | 0.999 | 3.09E-05 | 0.999 |  |  |  |  | 0.72 (0.53, 0.96) | 0.03 | cg13382100 | 1.04 (1.01, 1.07) | 0.01 |
|  | NBR1 |  | 1.08 (1.02, 1.15) | 0.01 | 0.880 | 7.56E-03 | 0.888 |  |  |  |  | 1.41 (1.06, 1.88) | 0.02 | cg05368731 | 0.99 (0.98, 1.00) | 7.83E-03 |
|  | PCBD2 |  | 1.17 (1.01, 1.36) | 0.04 | 1.000 | 3.77E-06 | 1.000 |  |  |  |  |  |  | cg05713859 | 0.97 (0.95, 1.00) | 0.04 |
